# Characteristics of the spiny dogfish (*Squalus acanthias*) nuclear genome

**DOI:** 10.1093/g3journal/jkad146

**Published:** 2023-07-03

**Authors:** C Isabel Wagner, Martina E L Kopp, James Thorburn, Catherine S Jones, Galice Hoarau, Leslie R Noble

**Affiliations:** Faculty of Biosciences and Aquaculture, Nord University, 8026 Bodø, Norway; Faculty of Biosciences and Aquaculture, Nord University, 8026 Bodø, Norway; School of Biology, University of St Andrews, St Andrews, KY16 9ST, UK; School of Applied Sciences, Edinburgh Napier University, Edinburgh, EH11 4BN, UK; School of Biological Sciences, University of Aberdeen, Aberdeen, AB24 3FX, UK; Faculty of Biosciences and Aquaculture, Nord University, 8026 Bodø, Norway; Faculty of Biosciences and Aquaculture, Nord University, 8026 Bodø, Norway

**Keywords:** *Squalus acanthias*, nuclear genome, de novo assembly, Selachii, shark

## Abstract

Sequenced shark nuclear genomes are underrepresented, with reference genomes available for only four out of nine orders so far. Here, we present the nuclear genome, with annotations, of the spiny dogfish (*Squalus acanthias*), a shark of interest to biomedical and conservation efforts, and the first representative of the second largest order of sharks (Squaliformes) with nuclear genome annotations available. Using Pacific Biosciences Continuous Long Read data in combination with Illumina paired-end and Hi-C sequencing, we assembled the genome de novo, followed by RNA-Seq-supported annotation. The final chromosome-level assembly is 3.7 Gb in size, has a BUSCO completeness score of 91.6%, and an error rate of less than 0.02%. Annotation predicted 33,283 gene models in the spiny dogfish's genome, of which 31,979 are functionally annotated.

## Introduction

Despite intense interest in sharks, many important areas of their biology remain largely unexplored. Although biology is in the era of genomics, only twelve of the over 500 described shark species (Fricke *et al.* 2023, accessed 15.02.2023) have sequenced nuclear genomes, and of those only nine have genome annotation information connected to them (Read *et al.* 2017; Hara *et al.* 2018; Marra *et al.* 2019; Weber *et al.* 2020; Zhang *et al.* 2020; Rhie *et al.* 2021; Nishimura *et al.* 2022; Sayers *et al.* 2022; Stanhope *et al.* 2023).

We report the sequencing, assembly, and annotation of the thirteenth shark genome, that of the spiny dogfish (*Squalus acanthias*). This expands the number of shark orders with available genome annotation information from three to four out of nine. Furthermore, Squaliformes is the second-largest shark order, making the genome annotations of *S. acanthias* the closest related to the 140 species within that order (Fricke *et al.* 2023), which could facilitate genome research for all of them. In particular, this resource could assist annotation of the nuclear genome of *Squalus suckleyi* (Ebert *et al.* 2010), the publicly available nuclear reference genome of which (Sayers *et al.* 2022) awaits annotation.

*S. acanthias*, a medium sized shark, occupies all oceans except for the North Pacific (Ebert *et al.* 2010). It has attracted interest from a biomedical perspective [e.g. as a source of the antibiotic squalamine (Moore *et al.* 1993)]. Furthermore, it was once dubbed possibly the most abundant extant shark but has suffer rapid and well documented, fisheries-induced population declines (Compagno 1984; Ellis *et al.* 2015, 2016; Finucci *et al.* 2020). Conservation of this species will benefit from better understanding and characterization of markers for genomic regions, enabling more direct associations between gene function and environmental parameters. Therefore, we anticipate genome characterization will advance scientific endeavors in these and other areas, allowing further genomic exploration and conservation of this species.

To sequence the genome of *S. acanthias*, we non-lethally sampled skin, muscle, and blood from a female in the North-East Atlantic. We then employed Pacific Biosciences (PacBio) Continuous Long Reads (CLRs) in combination with Illumina paired end (PE) and Hi-C sequencing for de novo assembly, followed by annotation using publicly available transcriptome datasets (Chana-Munoz *et al.* 2017). From this, we generated a high-quality, annotated draft genome, which allowed a first view of the unique characteristics comprising the nuclear genome of *S. acanthias*.

## Material and methods

### Sampling

A female spiny dogfish (total length 71 cm) was caught by rod and line with a baited, barbless hook in the Lynn of Lorn, UK, at 56°28′22″N 5°25′30″W, August 2019. Two tissue samples (muscle and skin) of ø 5 and 2 ml of whole blood, split into two 1.3 ml lithium heparin tubes, were sampled. All samples were immediately flash frozen in liquid nitrogen; subsequent storage was between −78.5 and −80°C. The individual was released alive.

Sampling was conducted under the Animals (Scientific Procedures) Act 1986, Project License #P05E95C50.

### DNA extraction for PacBio and Illumina short read sequencing

High molecular weight (HMW) DNA was extracted from frozen whole blood with the MagAttract HMW DNA kit (QIAGEN, Venlo, Netherlands), following 10× Genomics (Pleasanton, USA) recommendation with additional modifications and adjusted for the DNA content of nucleated blood cells in sharks (Saunders 1966; Hardie and Hebert 2003).

Separate extractions, each using 3, 5, 7.5, 10, 15, 20, 25, or 50 μl of whole blood, were performed. Briefly, whole blood was added to Proteinase K and mixed with RNase A and Buffer AL by pulse-vortexing. MagAttract Suspension G was then added to the mix, followed by Buffer MB. Two washing steps were performed with Buffer MW1, followed by two washing rounds with Buffer PE, and two rounds of washing with nuclease-free water. Final HMW DNA was eluted twice, first with 150 μl AE buffer, and again with 50 μl. Extracts were stored at −20°C and shipped on dry ice. See Supplementary File 1 for detailed protocol.

### Genome sequencing

Pacific Biosciences (PacBio) and short-read Illumina sequencing were performed by the Functional Genomics Laboratory and Vincent J. Coates Genomics Sequencing Laboratory, California Institute for Quantitative Biosciences (QB3), University of California, Berkeley, USA. PacBio CLR libraries were prepared with the SMRTbell Express Template Prep Kit 2.0, Sequel II Binding Kit 1.0 and Sequel II Internal Control Complex 1.0 and sequenced on two SMRTcells on a PacBio Sequel II machine (Pacific Biosciences of California, Inc., Menlo Park, USA).

For paired-end Illumina sequencing, DNA was fragmented on a Bioruptor Pico (Diagenode, Seraing, Belgium) and libraries prepared with the KAPA Hyper Prep kit for DNA (F. Hoffmann-La Roche AG, Basel, Switzerland), using six Polymerase Chain Reaction (PCR) cycles and a mean insert size of 430 bp. Library quality was evaluated on a Fragment Analyzer (AATI, now Agilent, Santa Clara, USA), molarity was assessed via quantitative PCR on a CFX Connect thermal cycler (BioRad, Hercules, USA), using the Kapa Biosystems Illumina Quant qPCR Kits (F. Hoffmann-La Roche AG, Basel, Switzerland). Libraries were pooled according to molarity and sequenced on a NovaSeq6000 150PE S4 flow cell (Illumina, Inc., San Diego, USA). The raw sequencing data was transferred into fastq-format via bcl2fastq2 (v. 2.20, Illumina Inc. 2019).

Hi-C sequencing was performed by the Norwegian Sequencing Centre, University of Oslo, Oslo, Norway. Two libraries were prepared from 0.08 g muscle tissue with the Dovetail Omni-C kit and Omni-C proximity Ligation Assay (v. 1.0, Dovetail Genomics, Scotts Valley, USA), and sequenced on an Illumina NovaSeq 150PE S4 flow cell (Illumina, Inc., San Diego, USA).

### Genome size estimation

Size of the nuclear genome was estimated with Jellyfish (v. 2.2.8, Marçais and Kingsford 2011) in combination with GenomeScope (online v. 1.0, Vurture *et al.* 2017).

Paired-end Illumina reads were first trimmed for adapters and low quality base calls with Trimmomatic (v. 0.39, Bolger *et al.* 2014), in PE mode for internally provided TruSeq3-PE-2 adapters. Seed mismatches were set to 2, palindrome and simple clip threshold to 30 and 10, respectively, and the minimum adapter length to be removed set to 1 bp, with both reads being retained after adapter trimming. Trimming of read ends was performed with a quality threshold of 3, followed by sliding window trimming with a window size of 4 bp, and a required base quality of 15. Next, reads were trimmed for poly-G tails, with cutadapt (v. 2.10, Martin 2011), with a phred threshold of 20, and a minimum read length of 1 bp to be retained.

The trimmed, paired reads were then fed to Jellyfish to count canonical 21-mers using a hash size of 140.961 Gbp, followed by construction of a count histogram with a maximum count value of 10,000,000. GenomeScope then used the histogram, adjusted for 21-mers, a read length of 151 bp, and a maximum k-mer coverage of 1,000,000, to model the genome size.

As organellar sequences can confound nuclear genome size estimates (Vurture *et al.* 2017), in a second approach the trimmed reads were first mapped to the mitochondrial genome of *S. acanthias* (Rasmussen and Arnason 1999; Sayers *et al.* 2022) as well as Phi X control sequences (Illumina, Inc., San Diego, USA). To allow mapping at both ends of the circular mitochondrial genome, the first 151 bases of the mitochondrial, fasta-formated sequence were duplicated at its opposite end. Read pairs were then mapped with BWA-MEM (v. 0.7.17-r1188, Li 2013); mapped reads as well as their respective read mate were discarded using SAMtools fastq (flag -f 13, v. 1.14, Danecek *et al.* 2021). Genome size was then modeled following the procedure described above.

### Assembly

The assembly process followed a modified version of the DNAnexus VGP assembly pipeline (v. 1.6), by Rhie *et al.* 2021. In the first step, raw PacBio subreads were assembled using the long-read assemblers Canu, Flye, and wtdbg2 (Koren *et al.* 2017; Kolmogorov *et al.* 2019; Ruan and Li 2020).

Canu assembler (v. 2.0, Koren *et al.* 2017) was adjusted for an estimated genome size of 5.7 Gb (Hardie and Hebert 2003). Longest reads were corrected and used up to a genome coverage of 200. Bogart was used for unitig constriction, with allowed standard deviations of read dissimilarity set to 3 for contig construction and bubble detection; and to 1 for repeat detection. Furthermore, heuristics for contig construction at repeats with multiple possible paths in the assembly graph were set to require a minimum of 500 bp or 50% larger overlap in the chosen path than in alternative paths.

For wtdbg2 (v. 2.5, Ruan and Li 2020), the estimated genome size was again set to 5.7 Gb. Following the authors’ recommendation, only the longest subreads were used, and all reads shorter than 5,000 bp discarded. Consensus was called with wtpoa-cns (v. 2.5), part of wtdbg2. Flye (v. 2.8-b1674, Kolmogorov *et al.* 2019) was run with default settings for raw PacBio CLR data. The assemblies generated with Flye and Canu were chosen for downstream analysis.

### Purging haplotypes

The assemblies produced by Canu and Flye were purged for uncollapsed haplotigs, using purge_dups (v. 1.2.5, Guan *et al.* 2020) in combination with minimap2 (v. 2.17-r941, Li 2018).

The purge_dups pipeline was run manually step by step, with a RAM threshold of 10 Gbp for minimap2. The assembly produced by Flye was purged in one round, with manually set cutoffs for the lower, middle, and upper read depth bounds of 5, 43, and 255, respectively. The Canu-derived assembly was purged in two consecutive rounds, first with manually set lower, middle, and upper bounds for read depths of 5, 21, and 126, respectively. In the second round, automatic cutoffs were used.

### First scaffolding

Illumina-derived Hi-C reads were used for scaffolding the primary assembly using the Arima-HiC Mapping Pipeline (v. 02, https://github.com/ArimaGenomics/mapping_pipeline, Arima Genomics, Inc., San Diego, USA) and Salsa (v. 2.3, Ghurye *et al.* 2017, 2019).

PE reads were first trimmed with Trimmomatic (v. 0.39, Bolger *et al.* 2014), followed by cutadapt (v. 3.4, Martin 2011, settings see Genome size estimation). Briefly for mapping, reads were first aligned with BWA-MEM (v. 0.7.17, Li 2013), followed by filtering of chimeric reads, pairing of read pairs, and filtering for a mapping quality threshold of 10. PCR duplicates were removed using Picard (v. 2.26.2, Broad Institute 2019). Lastly, the mapped reads of both libraries were merged, before scaffolding with Salsa, with settings for Omni-C data (Ghurye *et al.* 2017, 2019).

### Polishing

Scaffolds were polished with long-read data via Arrow (Chin *et al.* 2013), followed by polishing with Illumina short read data with Pilon (v. 1.24, Walker *et al.* 2014), one round each.

PacBio reads were aligned to the assembly with pbmm2 (v. 1.7.0, SMRT Link v. 10.2, Pacific Biosciences of California, Inc., Menlo Park, USA, Li 2018), and then used for polishing the assembly with the Arrow algorithm implemented in gcpp (v. 2.0.2, Pacific Biosciences of California, Inc., Menlo Park, USA, Chin *et al.* 2013). Previously trimmed Illumina reads (see Genome size estimation) were then mapped to the pre-polished assembly with BWA-MEM (v. 0.7.17, Li 2013), and used by pilon (Walker *et al.* 2014) to polish the assembly a second time, in diploid mode with manually assigned blocks. Each block used the read-mapping to the whole genome, but polished only sub-parts of the assembly, overcoming the issue of single-threading in pilon.

### Contamination filtering

The polished assembly was filtered for possible contaminants of foreign species and mitochondrial genomes in a three-step approach, using BLAST+ (v. 2.12.0, Altschul *et al.* 1990; Camacho *et al.* 2009).

First, all scaffolds were submitted to a nucleotide-nucleotide search optimized for highly similar matches (megablast) against the NCBI nucleotide database (nt, accessed 18.01.2022, Sayers *et al.* 2021), limited to hits that passed an expectation value threshold of 1e-4, and a maximum of five target sequences to be retained in the output. In a second round, all scaffolds without a hit in the previous search were submitted to another nucleotide-nucleotide search, this time optimized for somewhat similar matches (blastn, database: nt). As before, hits were limited to those that passed an expectation value threshold of 1e-4, with a maximum of five target sequences retained. All scaffolds with a hit outside the class Chondrichthyes were subsequently removed from the data set.

To filter for possible mitochondrial genomes contained in the assembly, all surviving scaffolds were submitted to a nucleotide-nucleotide search optimized for highly similar matches (megablast) against the mitochondrial reference genome of *S. acanthias* (Rasmussen and Arnason 1999), again filtered for matches that passed an expectation value threshold of 1e-4. Any scaffolds of completely mitochondrial origin were discarded.

### Second scaffolding

Following polishing and decontamination of the scaffolded assembly, the new scaffolding tool YaHS emerged (Zhou *et al.* 2023), and was therefore used to re-scaffold the polished and decontaminated scaffolds produced with Salsa (Ghurye *et al.* 2017, 2019), which had not reached chromosome-level lengths. The Salsa-derived scaffolds were re-scaffolded using the same trimmed Illumina-derived Hi-C reads as in the first scaffolding, which were mapped to the scaffolds with BWA-MEM (v. 0.7.17, Li 2013), then cleaned and merged using the Arima-HiC Mapping Pipeline (v. 02, https://github.com/ArimaGenomics/mapping_pipeline, Arima Genomics, Inc., San Diego, USA) as described before. Scaffolds were finally re-scaffolded with YaHS (v. 1.2a.1, Zhou *et al.* 2023). A Hi-C contact map was generated with Pre from Juicer Tools (version distributed with YaHS and stand-alone version 2.13.06, Durand, Shamim, *et al.* 2016), and visualized with Juicebox (v. 1.11.08, Durand, Robinson, *et al.* 2016).

### Annotation

#### Repeat masking

The YaHS-derived spiny dogfish genome assembly was soft-masked with RepeatMasker (v. 4.1.2-p1, Altschul *et al.* 1990; Benson 1999; Camacho *et al.* 2009; Smit *et al.* 2015), using species-specific repeat libraries from the Extensive de novo TE Annotator pipeline (EDTA, v. 2.0.0, Xu and Wang 2007; Ellinghaus *et al.* 2008; Xiong *et al.* 2014; Ou and Jiang 2018, 2019; Ou *et al.* 2019; Shi and Liang 2019; Su *et al.* 2019; Zhang *et al.* 2022) and RepeatModeler (v. 2.0.3, Benson 1999; Bao and Eddy 2002; Price *et al.* 2005; Flynn *et al.* 2020), combined with two short interspersed nuclear elements (SINEs) previously identified in higher elasmobranchs or *S. acanthias* itself (Ogiwara *et al.* 1999; Nishihara *et al.* 2006).

In preparation of repeat library construction, coding DNA sequences (CDS) were identified via genome-guided transcriptome assemblies. Transcriptome data derived from four tissues (brain, liver, kidney, and ovary), previously published by Chana-Munoz *et al.* (2017, retrieved 14.02.022 from the European Nucleotide Archive, Cummins *et al.* 2022), was analyzed for quality and adapter contamination via FastQC (v. 0.11.9, Babraham Bioinformatics 2010), and then adapter and quality trimmed via fastp (v. 0.23.2, Chen *et al.* 2018). In fastp, first read correction was conducted by PE read overlap. Low quality bases at the 5’ end of the read were dropped, with a phred score threshold of 20 within a 4 b sliding window. Following this, read pruning was conducted, starting again from the 5’ end with a phred score threshold of 20 within a 4 b sliding window, dropping the right part of the read if base quality sank below the set threshold. Adapters (automatically detected, Nextera, TruSeq2 and TruSeq3 PE) were trimmed as well as poly-X tails, and finally reads were filtered for a minimum length of 2 bp.

Next, trimmed reads, paired as well as unpaired, were mapped to the Salsa-scaffolded genome with HISAT2 (v. 2.2.1, Kim *et al.* 2015, 2019). Each tissue-specific data set was individually mapped with settings for downstream transcriptome assembly, in a non-deterministic manner, and sorted via SAMtools (v. 1.14, Danecek *et al.* 2021). Genome-guided, tissue-specific transcriptome assemblies were then conducted and combined using StringTie2 (v. 2.2.1., Pertea *et al.* 2015), with a minimal transcript length threshold of 30 bp for initial assembly construction, followed by merging of the four assemblies with default parameters. Finally, CDS were extracted via the TransDecoder pipeline (v. 5.5.0, Haas 2018), in default mode.

CDS were fed to EDTA (v. 2.0.0, Xu and Wang 2007; Ellinghaus *et al.* 2008; Xiong *et al.* 2014; Ou and Jiang 2018, 2019; Ou *et al.* 2019; Shi and Liang 2019; Su *et al.* 2019; Zhang *et al.* 2022) for purging of gene sequences from a repeat library produced in default mode. A second species-specific repeat library was constructed using RepeatModeler (v. 2.0.3, Benson 1999; Bao and Eddy 2002; Price *et al.* 2005; Flynn *et al.* 2020), using seven rounds and sampling 1.1 Gb of the genome for repeat detection. This time, protein coding sequences were purged by querying the sequences against the UniProtKB/Swiss-Prot database (accessed: 30.5.2022, The UniProt Consortium 2021) in a translated-nucleotide to protein search with BLAST+ (blastx, v. 2.12.0, Altschul *et al.* 1990; Camacho *et al.* 2009) with an *e*-value threshold of 1e-3, and removal of aligning sequences from the repeat library.

Both libraries were combined, and two known SINEs were added (Ogiwara *et al.* 1999; Nishihara *et al.* 2006). “SacSINE1” from Nishihara *et al.* is species-specific for *S. acanthias,* but as the sequence of SINE “HE1” from Ogiwara *et al.* is not, it was queried against the NCBI nucleotide database via the nucleotide-nucleotide BLAST+ web interface (blastn, nt data base accessed: 05.04.2022, Altschul *et al.* 1990; Johnson *et al.* 2008; Camacho *et al.* 2009; Sayers *et al.* 2021) with default settings. One sequence matching in *S. acanthias* was then included in the repeat library. The final repeat library was implemented for soft masking the YaHS-derived *S. acanthias* genome assembly with RepeatMasker (Altschul *et al.* 1990; Benson 1999; Camacho *et al.* 2009; Smit *et al.* 2015), run in sensitive mode, using NCBI BLAST+ modified for RepeatMasker as the search engine and omitting the masking of low complexity DNA and simple repeats.

#### Gene prediction

BRAKER2 was used for gene prediction in the YaHS-scaffolded genome assembly, using both RNA-Seq and protein evidence (v. 2.1.6, Lomsadze *et al.* 2014, 2005; Stanke *et al.* 2006, 2008; Gotoh 2008; Li *et al.* 2009; Barnett *et al.* 2011; Iwata and Gotoh 2012; Buchfink *et al.* 2015; Hoff *et al.* 2016, 2019; Brůna *et al.* 2020, 2021), followed by TSEBRA (v. 1.0.3, Gabriel *et al.* 2021) to combine the results of different gene prediction approaches.

For support with RNA-Seq data, transcriptome data from Chana-Munoz *et al.* (2017), corrected and trimmed as described earlier, was tissue-specifically mapped to the soft masked genome with HISAT2 (v. 2.2.1, Kim *et al.* 2015, 2019) and sorted with SAMtools (v. 1.14, Danecek *et al.* 2021). BRAKER2 was then run with the combined data as input, skipping all parameter training and using the human BRAKER2 pre-trained parameter set. The human parameter set was chosen because parameter sets trained specifically for *S. acanthias,* or provided by BRAKER2 for other organisms more closely related to our target species than *Homo sapiens,* resulted in much lower BUSCO gene set completeness for the finally predicted gene set. In a second approach, the Vertebrata section of OrthoDB v10 (Kriventseva *et al.* 2019), modified by declaring all selenocysteines to be amino acids of unknown identity, was used by BRAKER2 as protein evidence, again with the human parameter set and skipping all parameter training. In a third approach, BRAKER2 was run combining the RNA-Seq alignments plus the protein evidence from the two previous runs, again with the human parameter set, skipping all parameter training.

All three approaches were then amalgamated in various combinations via TSEBRA (Gabriel *et al.* 2021), using either default parameters which exclude all genes predicted without extrinsic supporting evidence, or with developer-provided configuration parameters that also retain ab initio predicted genes. However, after evaluation of gene set completeness with BUSCO (v. 5.2.2, Manni *et al.* 2021), the initial BRAKER2 run with RNA-Seq evidence only showed the highest completeness and was thus chosen for downstream analysis.

#### Functional annotation

For functional annotation, proteins predicted by BRAKER2 (v. 2.1.6, Lomsadze *et al.* 2014, 2005; Stanke *et al.* 2006, 2008; Gotoh 2008; Li *et al.* 2009; Barnett *et al.* 2011; Iwata and Gotoh 2012; Buchfink *et al.* 2015; Hoff *et al.* 2016, 2019; Brůna *et al.* 2020, 2021) were queried against the vertebrata UniProtKB/Swiss-Prot database (accessed: 19.4.2023, The UniProt Consortium 2021) in a protein to protein search with BLAST+ (blastp, v. 2.13.0, Altschul *et al.* 1990; Camacho *et al.* 2009) with an *e*-value threshold of 1e-6 and the output restricted to the maximum of a single target sequence and High Scoring Pair per query. Furthermore, the annotated protein sequences were queried against the InterPro data base (accessed: 20.4.2023, Blum *et al.* 2021) with InterProScan (v. 5.61-93.0, Jones *et al.* 2014), with the precalculated match lookup service disabled, but including the lookup of Gene Ontology and Pathway annotations. Biological information was then attached to genome features with the script agat_sp_manage_functional_annotation.pl in AGAT (v. 1.0.0, Dainat 2022).

### Evaluation

Sequence statistics [assembly size, N50 (the weighted median length of the assembled sequence length), fragment number, and the length of the longest fragment], sequence completeness levels and error rates of the different stages of the genome assembly were assessed via a custom python3 script using the Biopython package (Supplementary File 2, Python3 v. 3.8.5, Biopython v. 1.78, Cock *et al.* 2009), via BUSCO (v 4.1.4 - 5.2.2, Manni *et al.* 2021) and via Merqury involving meryl (both v. 1.3, Miller *et al.* 2008; Koren *et al.* 2017; Rhie *et al.* 2020). For the predicted gene sets, completeness was assessed via BUSCO only (v. 5.2.2, Manni *et al.* 2021).

BUSCO was run in genome mode with the vertebrata reference gene set (vertebrata_odb10, *n* = 3,354, Manni *et al.* 2021). In Merqury, the most appropriate k-mer size was determined for a potential genome size of 2.0 Gb (haploid), 11.0 Gb (diploid), and 14.4 Gb (diploid). A custom 21-mer database was then built from Illumina data trimmed as described earlier (“Genome size estimation”), using meryl (v. 1.3, Miller *et al.* 2008; Koren *et al.* 2017; Rhie *et al.* 2020), and counting the occurrence of canonical 21-mers for each data set individually before merging by summing them. Merqury was then run with the same meryl database for all stages of the genome assembly, in default mode.

For the predicted gene sets, completeness was assessed via BUSCO (v. 5.2.2, Manni *et al.* 2021), in protein mode with the vertebrata reference gene set (vertebrata_odb10, *n* = 3,354).

## Results and discussion

Genome sequencing provided a total of 297.9 Gb of PacBio CLR data, 716.3 Gb Illumina PE data and 589.2 Gb Hi-C data, covering the genome 71 times, 171 times, and 141 times, respectively (Table 1), based on an estimated genome size of 4,178,143,881 bp (see below).

**Table 1.** Sequencing data generated to assemble the nuclear genome of *Squalus acanthias*. Pacific Biosciences (PacBio) Continuous Long Reads (CLRs) were used in combination with Illumina paired end (PE) and Hi-C sequencing. Data characteristics were derived via a custom Python3 script (Supplementary File 2). N50 describes the weighted median length of the assembled sequence length. Genome coverage was calculated based on a genome size of 4,178,143,881 bp (see below).

Data type	Total data [b]	Sequence number	N50 [b]	Maximum sequence length [b]	Genome coverage
PacBio CLR	297,896,106,484	15,790,308	33,006	193,081	71.30
Illumina PE	716,327,706,496	4,743,892,096	151	151	171.45
Hi-C	589,216,490,262	3,902,095,962	151	151	141.02

Genome size estimation varied only marginally between the two computational approaches presented here, with a size of 4,178,143,881 bp for the data excluding the mitochondrial genome of the spurdog as well as potential Phi X contamination. The full data generated a genome size estimate of 4,178,415,829 bp, only 271,948 bp larger than the cleaned data set. Therefore, we conclude that the genome of the spiny dogfish should be around 4.18 Gb in size.

The reported genome is rich in repetitive regions, with both estimates by GenomeScope reporting a uniqueness of 36.9%, and RepeatMasker concordantly masking over 70% of the genome. Heterozygosity was estimated to a rate of 0.632%, again in both estimates by GenomeScope.

Assembling the genome with three different assemblers gave results of varying quality and quantity (Table 2). The Canu assembly was the largest (8.4 Gb), followed by that of wtdbg2 (5.2 Gb) and Flye (3.9 Gb), making the Canu assembly more than twice than that of Flye. However, when compared to a benchmarked reference set of single-copy genes in vertebrates (BUSCO, Seppey *et al.* 2019; Manni *et al.* 2021), duplication levels were comparable between wtdbg2 and Flye (<2%), whereas Canu had a duplication level of over 50% according to BUSCO scores. This can be attributed to the different approaches taken by the assemblers: wtdbg2 and Flye usually collapse haplotypes, whereas Canu was run trying to separate the two haplotypes.

**Table 2.** Characteristics of three assemblies for the nuclear genome of *Squalus acanthias*. N50 describes the weighted median length of the assembled sequence length, BUSCO scores are C(omplete) and S(ingle), C(omplete) and D(uplicated), F(ragmented) and M(issing).

Assembly	Assembly size	N50	Longest fragment	Fragment number	BUSCO scores	Per-base error rate
**wtdbg2, raw**	5.2 Gb	0.2 Mb	7.3 Mb	85,859	C: 74.5% [S: 72.9%, D: 1.6%], F: 10.7%, M: 14.8%	1.20%
**Flye, raw**	3.9 Gb	1.3 Mb	10.8 Mb	37,143	C: 85.7% [S: 83.8%, D: 1.9%], F: 6.2%, M: 8.1%	0.05%
**Canu, raw**	8.4 Gb	0.3 Mb	19.4 Mb	54,009	C: 83.7% [S: 33.6%, D: 50.1%], F: 7.6%, M: 8.7%	0.03%
**Flye, purged**	3.6 Gb	1.5 Mb	10.8 Mb	19,423	C: 85.6% [S: 83.8%, D: 1.8%], F: 6.4%, M: 8.0%	0.04%
**Canu, purged**	3.7 Gb	1.4 Mb	19.4 Mb	10,017	C: 82.7% [S: 81.2%, D: 1.5%], F: 8.4%, M: 8.9%	0.02%

The Canu and Flye assemblies had comparable BUSCO completeness levels of over 80%, whereas the wtdbg2 output has a completeness score of <75%. Nevertheless, N50 scores (the weighted median length of the assembled sequence length) were comparable between Canu (0.3 Mb) and wtdbg2 (0.2 Mb), and clearly surpassed by the Flye assembly (1.3 Mb). The Canu assembly can be expected to contain many poorly assembled genome fragments of the alternative haplotype, degrading its apparent success. Furthermore, it contained the longest contig of all three assemblies (19.4 Mb), suggesting a rather successful assembly. Finally, per-base error rates estimated by Merqury were lowest in Canu (0.03%) and Flye (0.05%), when compared to wtdbg2 (1.20%). Therefore, considering all assembly characteristics, both the Canu- and Flye-derived assemblies were chosen for further processing.

Purging of haplotigs led to an increase in some assembly quality parameters for both assemblies, but decreased others. The error rates improved by 0.01% for both assemblies, and the N50 increased by 1.1 Mb for the Canu assembly and 0.2 Mb for the Flye assembly. Duplication levels sank below 2%, for the Flye assembly after one round and for the Canu assembly after two rounds of purging. However, the rate of complete BUSCOs decreased for both assemblies, more strongly for the Canu (1.0%) than for the Flye assembly (0.1%). In both cases, parts of this can be explained by an increase of fragmented BUSCOs, however, the Canu assembly lost true genomic information during purging, as can be seen from an increase (0.2%) of missing BUSCOs.

In total, after purging the Canu assembly had lower error and genome duplication rates than the Flye assembly but was surpassed by the Flye assembly with a higher N50 and BUSCO completeness score. As a higher quality assembly, with lower duplication levels and error rates, should benefit the scaffolding process, the Canu assembly was selected for downstream analysis.

**Fig. 1.**
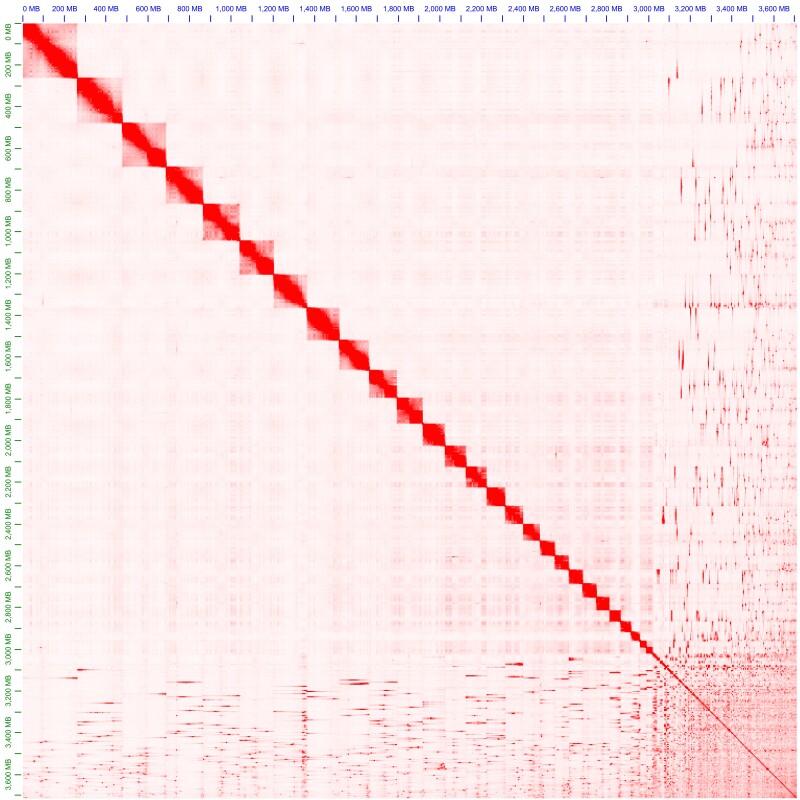
Hi-C contact map for the nuclear genome assembly of *Squalus acanthias*, scaffolded with YaHS (Zhou *et al*. 2023). Total assembly length is 3.7 Gb. The map was visualized with Juicebox (v. 1.11.08, Durand *et al*. 2016a).

Scaffolding of the Canu assembly with Hi-C data and Salsa (Ghurye *et al.* 2017, 2019) increased the rate of complete BUSCOs to over 90%, and the sequence N50 from 1.4 Mb to 10.5 Mb (Table 3). However, only 1.3 Gb of the assembly were contained in the 30 and 31 longest scaffolds, the expected haploid karyotype of *S. acanthias* (Nygren *et al.* 1971; Nygren and Jahnke 1972; Schwartz and Maddock 1986; but see Stingo and Rocco 2001). The scaffolded and polished assembly, cleared from 417 scaffolds containing foreign organism contamination and one scaffold completely of mitochondrial origin, was scaffolded a second time with the tool YaHS (Zhou *et al.* 2023). This time, the assembly reached an N50 of 124.1 Mb, and the 30 longest scaffolds accumulated to 3.07 Gb, 82.78% of the total assembly length (Fig. 1). Upon manual investigation of the Hi-C contact map (Fig. 1), one of the longest 30 scaffolds (“scaffold_29”, length: 13,596,185 bp) appears to be part of another larger scaffold (“scaffold_20”). We thus conclude that our final assembly reached pseudo-chromosomal level, identifying 29 out of 30 to 31 putative chromosomes, but can be improved further in the future.

**Table 3.** Characteristics of the nuclear genome assembly of *Squalus acanthias*. N50 describes the weighted median length of the assembled sequence length, BUSCO scores are C(omplete) and S(ingle), C(omplete) and D(uplicated), F(ragmented) and M(issing).

Assembly step	Assembly size	N50	Longest fragment	Fragment number	BUSCO scores	Per-base error rate
**Raw**	8.4 Gb	0.3 Mb	19.4 Mb	54,009	C: 83.7% [S: 33.6%, D: 50.1%], F: 7.6%, M: 8.7%	0.03%
**Purged**	3.7 Gb	1.4 Mb	19.4 Mb	10,017	C: 82.7% [S: 81.2%, D: 1.5%], F: 8.4%, M: 8.9%	0.02%
**Scaffolded with Salsa**	3.7 Gb	10.5 Mb	90.6 Mb	6,090	C: 90.7% [S: 88.3%, D: 2.4%], F: 4.0%, M: 5.3%	0.02%
**Polished with long-read data**	3.7 Gb	10.5 Mb	90.7 Mb	6,090	C: 91.0% [S: 88.6%, D: 2.4%], F: 3.9%, M: 5.1%	0.02%
**Fully polished**	3.7 Gb	10.5 Mb	90.6 Mb	6,090	C: 91.0% [S: 88.6%, D: 2.4%], F: 3.9%, M: 5.1%	< 0.02%
**Contamination-free**	3.7 Gb	10.7 Mb	90.6 Mb	5,672	C: 91.0% [S: 88.6%, D: 2.4%], F: 3.9%, M: 5.1%	< 0.02%
**Scaffolded with YaHS**	3.7 Gb	124.1 Mb	266.4 Mb	3,899	C: 91.6% [S: 89.2%, D: 2.4%], F: 3.7%, M: 4.7%	< 0.02%

Our final assembly has an N50 of 124.1 Mb, and is 91.6% complete according to BUSCO scores. The error rate is 38.07 in phred score or 0.01559%, according to Merqury.

RepeatMasker was used to soft-mask 73.79% of the genome (Supplementary File 3). Based on RNA-Seq evidence, BRAKER2 predicted a total of 37,280 genes in the masked genome, with a protein BUSCO completeness score of 88.8% (Complete and single copy: 72.3%, Complete and duplicated: 16.5%, Fragmented: 5.8%, Missing: 5.4%). High duplication levels can be attributed to multiple protein sequences per gene being included in the analysis. Protein evidence from other vertebrate genomes did not lead to higher gene set completeness (data not shown). Functional annotation attached biological information to 31,979 of these genes [Supplementary File 4 (raw results BLAST+) and Supplementary File 5 (raw results InterProScan)]. Together with the gene models that received full or partial RNA-Seq support during the structural annotation process (Supplementary File 6), this resulted in 33,283 gene models with external support. Due to their external support, we considered these (Supplementary File 7) to be more reliable than the rest of the gene model set. We acknowledge that our annotation approach can only be considered as a first version, as gene numbers are around 10,000–15,000 above what might be expected following the gene numbers found in high quality genome annotation of other shark species (especially Rhie *et al.* 2021; Sayers *et al.* 2022).

## Conclusion

We report the nuclear draft genome, and its annotation, of the spiny dogfish (*S. acanthias*). Together with the existing interest in this shark's biomedical characteristics, and its ecological importance, this assembled genome will facilitate further, more focused research on a variety of topics in this species. Furthermore, we expect that this resource will facilitate genomic research in other shark species, for example assisting reference-guided genome or transcriptome assemblies, or their annotation, as well as comparative genomics or phylogenomic analysis in other sharks.

## Supplementary Material

jkad146_Supplementary_Data

## Data Availability

For a detailed bench protocol for high-molecular weight DNA extractions and a Python3 script for assembly statistics see Supplementary Files 1 and 2. The raw sequencing data (Hi-C, PacBio CLR, and Illumina short reads) and final assembly can be found on NCBI under BioProject PRJNA978993. Repeat content information are included in Supplementary File 3, and annotation information in Supplementary File 8; further information regarding annotation can be found in Supplementary Files 4 to 7. Supplementary Files are available on the GSA figshare: https://doi.org/10.25387/g3.23260280. Supplemental material available at G3 online.
